# Supplementary material for: Curated and harmonised transcriptomics datasets of interstitial lung diseases
Source: Data Brief. 2025 Oct 14;63:112139. doi: 10.1016/j.dib.2025.112139 (PMC12581653; doi:10.1016/j.dib.2025.112139)
Supplement: Supplementary file 1 [file mmc1.zip › Supplementary_material/RNA-seq/fastQC reports/GSE184316/SRR15926746_fastqc.html]

SRR15926746.fastq.gz FastQC Report 

FastQC Report

Mon 5 Feb 2024  
SRR15926746.fastq.gz

## Summary

- Basic Statistics
- Per base sequence quality
- Per sequence quality scores
- Per base sequence content
- Per sequence GC content
- Per base N content
- Sequence Length Distribution
- Sequence Duplication Levels
- Overrepresented sequences
- Adapter Content

## Basic Statistics

| Measure | Value |
| --- | --- |
| Filename | SRR15926746.fastq.gz |
| File type | Conventional base calls |
| Encoding | Sanger / Illumina 1.9 |
| Total Sequences | 8743746 |
| Sequences flagged as poor quality | 0 |
| Sequence length | 25-382 |
| %GC | 49 |

## Per base sequence quality

## Per sequence quality scores

## Per base sequence content

## Per sequence GC content

## Per base N content

## Sequence Length Distribution

## Sequence Duplication Levels

## Overrepresented sequences

| Sequence | Count | Percentage | Possible Source |
| --- | --- | --- | --- |
| GGAAGACAGTGTGGCGATTCCTCAGGGATCTAGAACTAGAAATACCATTG | 153675 | 1.7575419048083054 | No Hit |
| ATTGATTCTTCCTACCCATGAGCATGGAATGTTCTTCCATTTGTTTGTAT | 107597 | 1.2305595336369561 | No Hit |
| GAAGGACCTCTTCAAGGAGAACTACAAACCACTGCTCAAGGAAATAAAAG | 102586 | 1.173250000629021 | No Hit |
| ATAGCAGCATGATTTATAGTCCTTTGGGTATATACCCAGTAATGGGATGG | 93622 | 1.070731011628197 | No Hit |
| GAAGGACCTCTTCAAGGAGAACTACAAACCACTGCTCAATGAAATAAAAG | 69809 | 0.7983877848235756 | No Hit |
| ATAGCAGCATGATTTATAATCCTTTGGGTATATACCCAGTAATGGGATGG | 45850 | 0.5243747931378611 | No Hit |
| ATTGATTCTTCCTACCCATGAGCATGGAATGTTCTTCCATTTGTTTGTGT | 26124 | 0.2987735462580912 | No Hit |
| GGAAGACAGTGTGGCGATTCCTCAAGGATCTAGAACTAGAAATACCATTG | 24573 | 0.28103515358291514 | No Hit |
| GGCCATTTTCATGATATTGATTCTTCCTACCCATGAGCATGGAATGTTCT | 21958 | 0.25112806341812766 | No Hit |
| GCATGTGTCTTTATAGCAGCATGATTTATAGTCCTTTGGGTATATACCCA | 21244 | 0.2429622269448358 | No Hit |
| GGAAGACAGTGTGGTGATTCCTCAGGGATCTAGAACTAGAAATACCATTG | 20610 | 0.23571133013241694 | No Hit |
| GGAAGACAGTGTGGCGATTCCTCAGGGATCTAGAACTAGAAATACCATTT | 18942 | 0.21663483820321405 | No Hit |
| ATAGCAGCATGATTTATAGTCATTTGGGTATATACCCAGTAATGGGATGG | 18192 | 0.2080572788825293 | No Hit |
| GGAAGACAGTGTGGCGATTCCTCAGGGATCTAGAACTGGAAATACCATTG | 17321 | 0.19809587332477407 | No Hit |
| GGAAGACAGTGTGGCAATTCCTCAGGGATCTAGAACTAGAAATACCATTG | 16697 | 0.19095934396996436 | No Hit |
| CACCTCCCCGAAGCTCAGGGAGAGCCCTGTTAGGGCCGCCTCTGGCCCTA | 14001 | 0.1601258773985429 | No Hit |
| ATTGATTCTTCCTATCCATGAGCATGGAATGTTCTTCCATTTGTTTGTGT | 13921 | 0.15921093773766987 | No Hit |
| CAGTGGGGCCACGAGCTGAGTGCGTCCTGTCACTCCACTCCCATGTCCCT | 13693 | 0.1566033597041817 | No Hit |
| AGGCTACACACTGAGGACTCTGTTCCTCCCCTTTCCGCCTAGGGGAAAGT | 13394 | 0.15318377272166872 | No Hit |
| GAAGGACCTCTTCAAGGAGAACTACAAACCACTGCTCAACGAAATAAAAG | 13257 | 0.15161693855242364 | No Hit |
| GGAAGACAGTGTGGTGATTCCTCAAGGATCTAGAACTAGAAATACCATTG | 13188 | 0.15082780309492064 | No Hit |
| GCATGTGTCTTTATAGCAGCATGATTTATAATCCTTTGGGTATATACCCA | 12463 | 0.1425361624182587 | No Hit |
| GGAAGACAGTGTGGCAATTCCTCAAGGATCTAGAACTAGAAATACCATTG | 11849 | 0.13551400052105814 | No Hit |
| ATTGATTCTTCCTACCCATGAGCATGGAATGTTCTTCCATTTCTTTGTAT | 10545 | 0.12060048404882759 | No Hit |
| CAATCCACTTGATCCCAACTCATCTCTCATTTATTTCGGCTTCTTTTATT | 10473 | 0.11977703835404185 | No Hit |
| TGATATGGTTCCTGGCAAGCCCATGTGTGTTGAGAGCTTCTCAGACTATC | 10227 | 0.11696359889685724 | No Hit |
| GGGAAGCGGGGAATGTCTACGTGCGTATGCACGTGGCACTCTCTGCCCGA | 9775 | 0.11179418981292458 | No Hit |
| TGATGACACCCACCGCAACTGTCTGTCTCATATCACGAACAGCAAAGCGA | 9390 | 0.10739104269497307 | No Hit |
| CCAACTCGGGGGCCTGGAGGCAGGGGTGGTCTCTCTTGAGCTCTCCTTGC | 9119 | 0.10429168459376564 | No Hit |
| TTACAAAAGCAGAATAAAAGCGAAAAGAAATGAAAATGTTACACTACATT | 8777 | 0.1003803175435334 | No Hit |

## Adapter Content

Produced by FastQC (version 0.11.7)
